# Remodeling of synaptome architecture following traumatic brain injury

**DOI:** 10.1016/j.isci.2026.118044

**Published:** 2026-10-29

**Authors:** Aimun A.B. Jamjoom, Ragini Gokhale, Jonathan Rhodes, Zhen Qiu, Seth G.N. Grant

**Affiliations:** 1 Genes to Cognition Programme, Institute for Neuroscience and Cardiovascular Research, University of Edinburgh, Edinburgh EH16 4SB, UK; 2 Department of Neurosurgery, Queen’s Hospital, Barking, Havering and Redbridge University Hospitals NHS Trust, London RM7 0AG, UK; 3 The Blizard Institute, Queen Mary University of London, London E1 2AT, UK; 4 Anaesthesia, Critical Care and Pain Medicine, Usher Institute, University of Edinburgh, Edinburgh EH16 4SA, UK; 5 Department of Biomedical Engineering, University of Strathclyde, Glasgow G4 0NW, UK; 6 Simons Initiative for the Developing Brain (SIDB), Centre for Discovery Brain Sciences, University of Edinburgh, Edinburgh EH8 9XD, UK; 7 Euan MacDonald Centre, University of Edinburgh, Edinburgh EH16 4SB, UK

**Keywords:** synaptome architecture, synapse diversity, traumatic brain injury, mouse brain atlas, postsynaptic scaffold proteins, synapse vulnerability, protein lifetime-dependent vulnerability

## Abstract

In the brain, excitatory synapse diversity is organized into a brainwide synaptome architecture. Traumatic brain injury is associated with synapse loss, but its impact upon synaptome architecture is unknown. Using synaptome mapping across 222 brain regions in mice, we show that lateral fluid percussion injury induces a progressive and distributed reorganization of excitatory synapse populations over 28 days. Although whole-brain synapse number is preserved, specific molecular types and subtypes of synapses exhibit region- and time-dependent vulnerability. At 7 days after injury, synapse types with rapid turnover of the postsynaptic scaffold protein PSD95 are preferentially reduced. However, by 28 days, they have increased, whereas those synapses with slow turnover are reduced. Microglial activation correlated with this synaptome remodeling in a temporally structured manner. These findings reveal a two-phase, proteostasis-linked reprogramming of synaptome architecture, suggesting that large-scale rebalancing of molecularly distinct synapse populations is a general mechanism underlying injury-induced brain plasticity.

## Highlights

• Brainwide synaptome mapping reveals synapse-specific remodeling in a mouse TBI model
• Injury causes delayed synapse loss in the contralateral cortex and hippocampus
• Short and long protein lifetime synapses show a biphasic response to injury
• Microglial activation correlates with synaptome remodeling over time

## Graphical abstract

**Figure:**
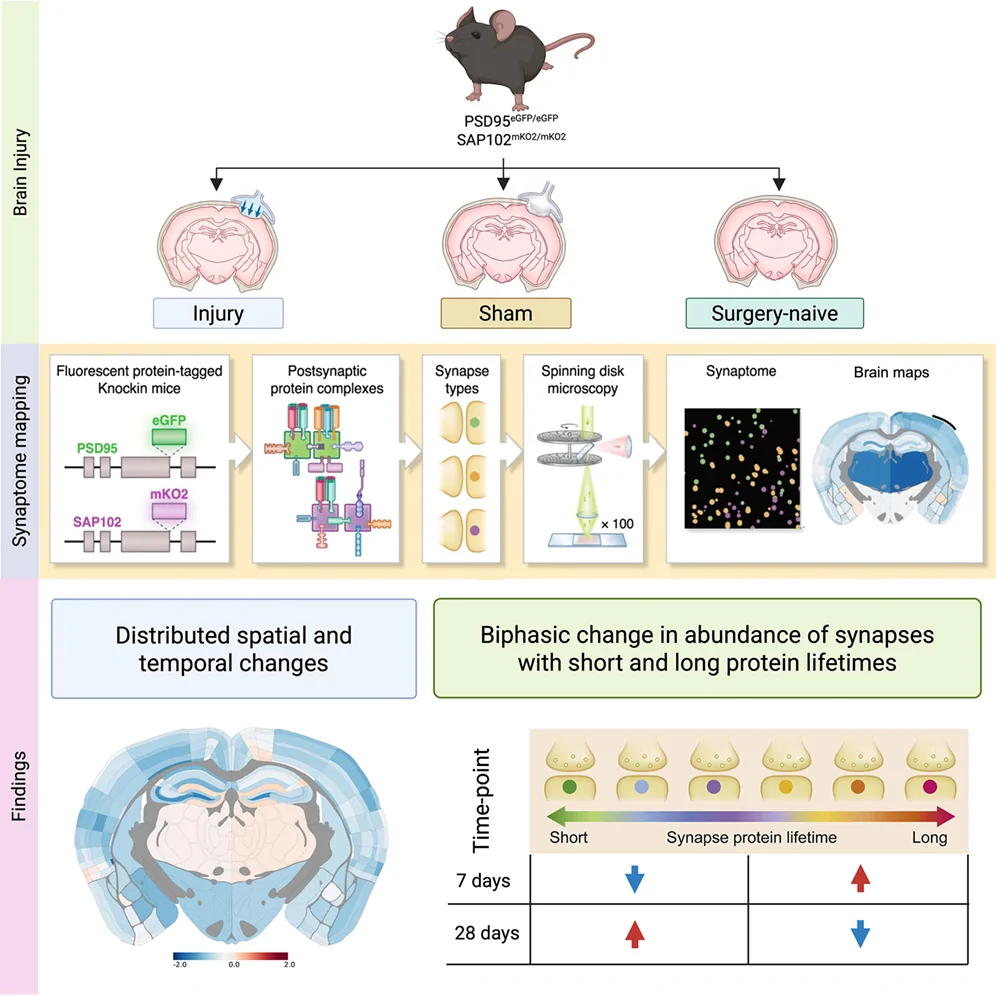

## Teaser

Biological sciences; Neuroscience; Cell biology

## Introduction

Traumatic brain injury (TBI) is a leading cause of mortality and long-term neurological disability worldwide.^1^ Although synapse loss is widely reported after injury, most studies have examined limited brain regions or small populations of synapses, emphasizing changes in dendritic spines or selected proteins.^2^^,^^3^^,^^4^^,^^5^^,^^6^ However, excitatory synapses are not uniform entities: they comprise a vast repertoire of molecularly distinct varieties that differ in composition, nanoarchitecture, and protein turnover.^2^^,^^7^^,^^8^^,^^9^^,^^10^^,^^11^^,^^12^ Brainwide synaptome mapping, which examines the composition, dynamics, and organization of synapse proteins at single-synapse resolution, has revealed that billions of excitatory synapses in the mouse brain are organized into a structured 3D architecture composed of discrete types and subtypes.^2^^,^^7^^,^^8^^,^^9^^,^^10^^,^^11^^,^^12^ This synaptome architecture undergoes systematic remodeling during development and aging,^7^^,^^8^^,^^9^ in response to sleep deprivation,^11^ sensory deprivation,^12^ and gene mutations, including those in genetic models of neuropsychiatric disease.^7^^,^^9^^,^^10^ Whether traumatic injury selectively disrupts defined synapse types and subtypes and reorganizes synaptome architecture remains unknown.

Here, we apply brainwide synaptome mapping to a lateral fluid percussion injury (LFPI) model to determine how TBI reshapes excitatory synapse populations across 222 anatomical regions of the mouse brain. By comparing LFPI with craniotomy alone (sham-LFPI) and analyzing synapse density, molecular composition, and protein lifetime classes at 7 and 28 days post-injury, we define the systems-level principles governing synaptic vulnerability and architectural remodeling after brain trauma. We further show that the synaptome response to injury shares conserved features with responses to sleep deprivation, genetic perturbation, and experience, revealing common adaptive mechanisms.

## Results

### Characterization of the LFPI model

Prior to the synaptome studies, the LFPI model was characterized in a cohort of wild-type male C57BL/6 mice (*n* = 15 injury, *n* = 11 sham; age 8–12 weeks). A fluid percussion pulse of 1.18 ± 0.03 atm was delivered. Injury mice showed a significantly elevated righting reflex time compared with sham mice (431.4 ± 178.2 vs. 40.3 ± 57.5 s; *p* < 0.0001, Mann-Whitney U test), consistent with a transient period of disrupted brain function. At 24 h post-injury, the mice were euthanized, and their brains were examined. Hematoxylin and eosin staining showed no overt cortical contusion or hippocampal neuronal cell death but revealed dysmorphic neurons within cortical layers 1 and 2/3 in all injury mice (extending into layer 6a in 43%) and ipsilateral corpus callosal fracturing in 85% of injury mice compared with 17% of sham mice (*p* = 0.01, chi-squared). Traumatic axonal injury, indexed by amyloid precursor protein (APP) immunohistochemistry, was prominent in the ipsilateral corpus callosum of injury mice (74.1 ± 38.6 APP-positive aggregates) and significantly elevated relative to both the contralateral corpus callosum of injury mice (1.8 ± 2.1; *p* < 0.0001) and the ipsilateral corpus callosum of sham mice (0.8 ± 1.0; *p* < 0.0001) (Figure S1A). Microglial (IBA1) (Figure S1B) and astrocytic (GFAP) (Figure S1C) immunostaining revealed a regional inflammatory response: IBA1 and GFAP cell counts were significantly elevated in the ipsilateral hippocampus of injury compared with sham mice (*p* = 0.0006 and *p* = 0.025, respectively), with additional GFAP elevation in the ipsilateral cortex of injury versus sham mice, while no significant differences were observed in the thalamus or hypothalamus. Together, these findings confirm that the LFPI protocol produces a reproducible mild injury characterized by transient functional impairment, diffuse axonal injury in the corpus callosum, and a regionally restricted neuroinflammatory response, in the absence of gross structural damage or overt neuronal loss.

### Experimental design and brainwide synaptome mapping after LFPI

We applied validated tools and protocols for brainwide synaptome mapping of excitatory synapse populations^7^^,^^8^^,^^9^^,^^10^^,^^11^ (Figure 1A). Transgenic mice expressing PSD95-eGFP and SAP102-mKO2 fusion proteins were used to fluorescently label postsynaptic scaffold proteins that assemble signaling complexes at excitatory synapses^13^^,^^14^^,^^15^ and are essential for synaptic plasticity and cognitive function.^16^^,^^17^^,^^18^ A group of mice (*N* = 21) received a single LFPI pulse (1.0–1.5 atm) delivered to the intact dura over the left parietal cortex; a second group (*N* = 18; sham-LFPI) received a craniotomy with intact dura but no fluid percussion; and a third group (*N* = 5) were surgery-naive (Figure 1A). LFPI and sham-LFPI groups were analyzed at 7 days and 28 days post-injury. These time points were selected to capture the subacute and chronic phases of the response to LFPI, respectively. They correspond to well-characterized stages of injury progression in both the LFPI model and the broader TBI literature, encompassing axonal injury, neuroinflammation, and early synaptic disruption, as well as longer term remodeling and plasticity.^4^^,^^19^^,^^20^

**Figure 1:**
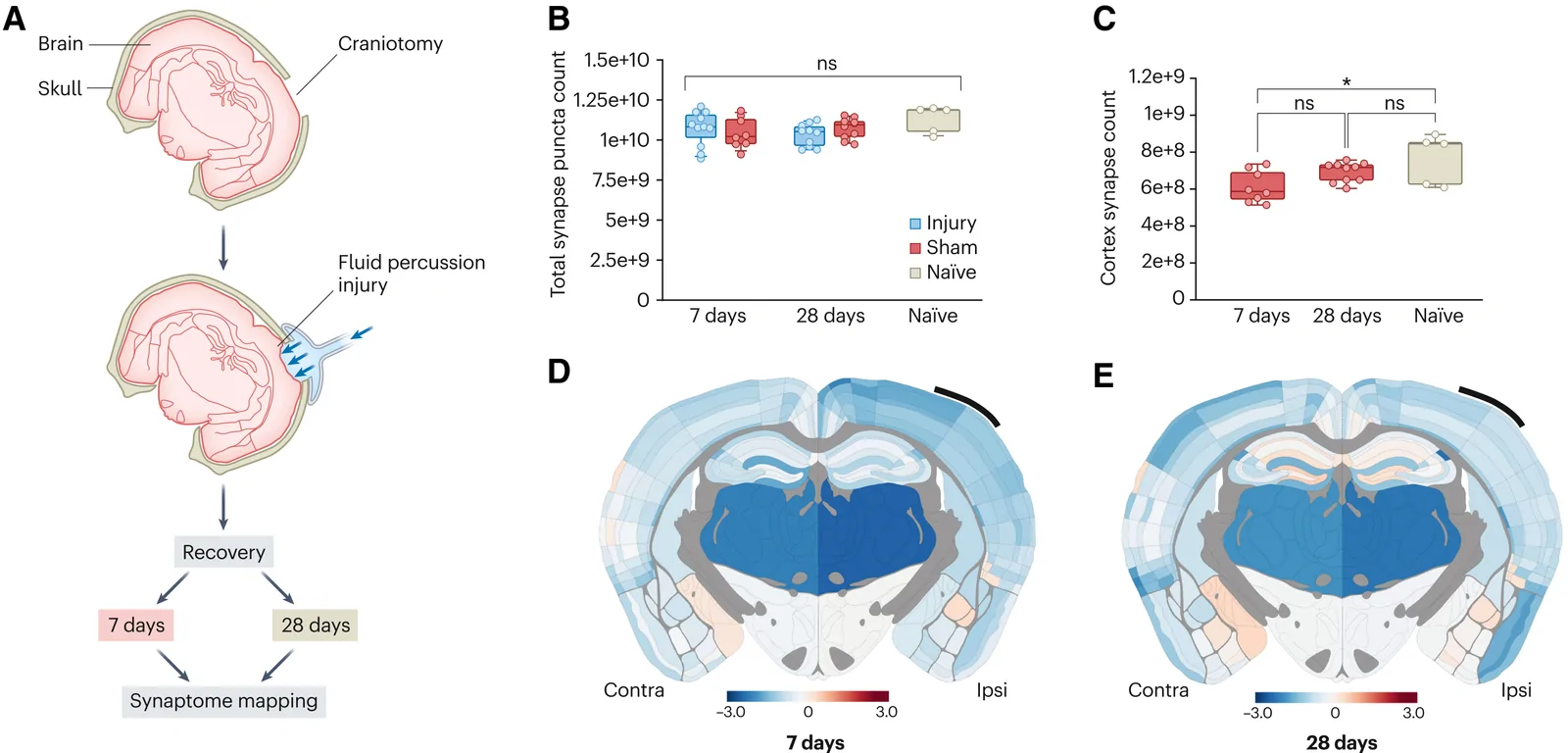
Impact of lateral fluid percussion injury on the mouse brain synaptome (A) Lateral fluid percussion injury (LFPI) experimental pipeline. (B) Comparison of whole-brain synapse puncta count across mouse cohorts (injury, sham-LFPI, and surgery-naive) and time points (7 and 28 days). Boxplots show the median (center line) and interquartile range (box limits, first to third quartile); whiskers indicate the 5th and 95th percentiles, and each point represents an individual mouse. ns, *p* > 0.05. (C) Comparison of synapse puncta count in the ipsilateral cortex underlying craniotomy between sham-LFPI and surgery-naive groups. Boxplots show the median (center line) and interquartile range (box limits, first to third quartile); whiskers indicate the 5th and 95th percentiles, and each point represents an individual mouse. ∗*p* < 0.05, one-way ANOVA with Tukey multiple comparisons test. (D and E) Spatial heatmaps of effect size (Cohen’s *d*) comparing sham-LFPI and surgery-naive groups at 7 days (D) and 28 days (E). Black line above cortex, injury site; Ipsi, ipsilateral hemisphere to injury; Contra, contralateral hemisphere to injury.

Mid-coronal brain sections at bregma −1.9 mm were imaged using spinning disc confocal microscopy at 100× magnification (optical resolution ∼260 nm; pixel size 84 nm) (Figure S2). Synaptic puncta were quantified in each mouse across 222 anatomically defined subregions (111 subregions in the ipsilateral and contralateral hemispheres). Images were processed with the SYNMAP synaptome mapping pipeline,^10^ which detects individual excitatory synapses and classifies them into three types: type 1 expressing only PSD95, type 2 expressing only SAP102, and type 3 expressing both PSD95 and SAP102. These three types were further subdivided into 37 molecularly and morphologically distinct synapse subtypes based on protein abundance, shape, and size (Figure S3). Of these, 30 subtypes were additionally stratified into short protein lifetime (SPL) and long protein lifetime (LPL) categories based on the previously defined PSD95 lifetime.^7^ Across all cohorts, the median whole-brain synapse puncta count (total number of synapse subtypes) was 1.075 × 10^10^ with an interquartile range of 1.41 × 10^9^ (Q1 = 9.87 × 10^9^, Q3 = 1.13 × 10^10^) (Data S11, S12, S13, S14, and S15). There was no statistical difference in the whole-brain puncta count across the cohorts at 7 and 28 days (*p* > 0.05; one-way ANOVA with Tukey multiple comparisons test) (Figure 1B).

### Craniotomy alone induces local and distributed synapse loss

Craniotomy is a widely used procedure in experimental neuroscience, including for the imaging of synapses, yet little is known about the effect of the procedure on synapses.^21^^,^^22^ We first compared sham-LFPI and surgery-naive groups to determine whether craniotomy alone affected excitatory synapse number. In the isocortex beneath the craniotomy (primary motor, secondary motor, and primary somatosensory areas), a significant reduction in total puncta count was observed at 7 days (*p* < 0.05; one-way ANOVA with Tukey’s test) but not at 28 days (Figure 1C; Data S12, S14, and S15). Brain maps of effect sizes (Cohen’s *d*) between sham-LFPI and surgery-naive groups revealed reduced synapse density within the thalamus at both 7 and 28 days, indicating that the impact of craniotomy extends to subcortical regions (Figures 1D and 1E; Data S12, S14, and S15). Thus, craniotomy alone, even with intact dura, can produce synapse loss in both local and distant brain regions.

### LFPI causes delayed and bilateral disruption of synaptome architecture

We next examined LFPI in comparison to surgery-naive mice. At 7 days, the greatest synapse loss was observed in isocortical regions underlying the injury site (*p* < 0.05, Bayesian estimation with Benjamini-Hochberg correction) (Figure S4; Data S1, S5, S6, and S10). By 28 days, puncta density was reduced across the isocortex and hippocampus in both hemispheres. Comparison of LFPI and sham-LFPI groups showed little difference at 7 days (*p* > 0.05, Bayesian estimation with Benjamini-Hochberg correction) (Figures 2A and 2C; Figure S5; Data S1, S2, S6, and S7). However, by 28 days, LFPI caused a significant decrease in the density of PSD95-expressing synapses in the contralateral isocortex and hippocampus (*p* < 0.05, Bayesian estimation with Benjamini-Hochberg correction) (Figures 2B and 2C; Data S4, S5, S8, and S9). In addition to changes in synapse density, LFPI altered the mean intensity (protein content) and size of PSD95- and SAP102-expressing synapses in multiple regions (Figure S5; Data S4, S5, S8, and S9). Together, these findings indicate that LFPI modifies the abundance and the molecular and structural properties of excitatory synapses across distributed brain regions.

**Figure 2:**
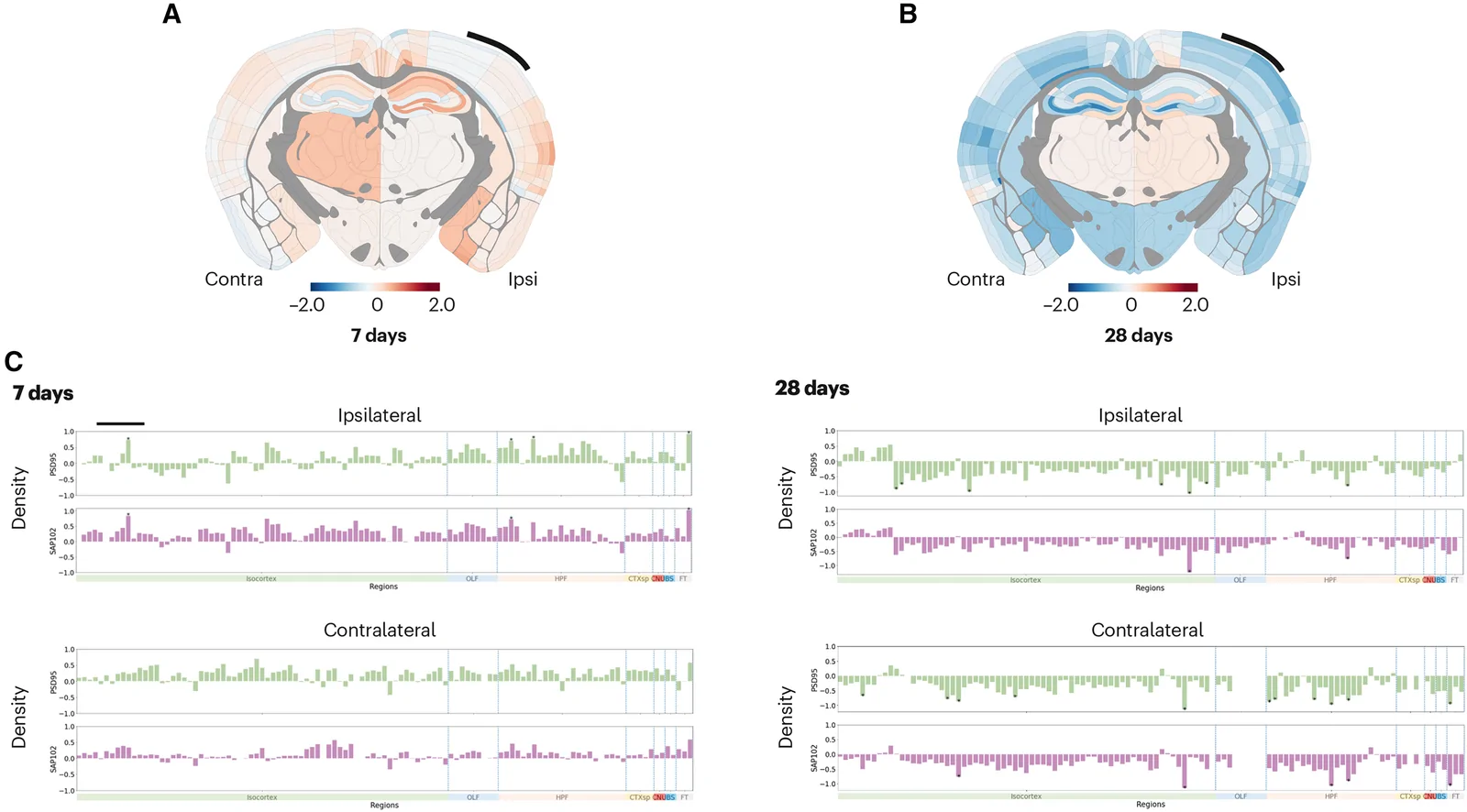
LFPI leads to delayed disruption of the synaptome that spreads to the contralateral hemisphere (A and B) Spatial heatmaps of effect size (Cohen’s *d*) comparing LFPI (injury) and sham-LFPI groups at (A) 7 days and (B) 28 days. Black line above cortex, injury site; Ipsi, ipsilateral hemisphere to injury; Contra, contralateral hemisphere to injury. (C) Difference between LFPI and sham-LFPI in PSD95 (green) and SAP102 (magenta) puncta density (Cohen’s *d* effect size) at 7 and 28 days. Expanded versions that include intensity and size are shown in Figure S5. ∗*p* < 0.05, Bayesian estimation with Benjamini-Hochberg correction.

### Injury induces type- and subtype-specific, anatomically distributed remodeling of synaptome architecture

To examine these injury-induced changes in greater detail, we generated heatmaps of effect size (Cohen’s *d*) comparing LFPI with sham-LFPI for each synapse type and subtype in 111 subregions in each hemisphere at 7 and 28 days (Figure S6; Data S11, S12, S13, and S14). LFPI caused some subtypes to increase and others to decrease in density in a highly distributed manner at both time points. For example, type 1 subtypes 2–5 were decreased in cortical areas near the injury site at 7 days and showed widespread reduction across brain regions in both hemispheres by 28 days. Type 2 subtypes 12–15 were decreased in both ipsilateral and contralateral cortices at 7 and 28 days, suggesting particular vulnerability. Type 3 subtypes 20–31 were reduced at 7 days but increased at 28 days in the ipsilateral cortex. Within individual regions, some subtypes increased while others decreased, indicating redistribution among molecularly defined synapse populations rather than uniform loss. These results show that brain injury induces local and distributed changes in the density of specific synapse types and subtypes that evolve over time.

### Differential vulnerability of synapse subtypes defined by protein lifetime

The dynamic evolution of synaptome architecture that we observed following brain injury may inform mechanisms of synaptic resilience and repair. We hypothesized that the changes in synapse molecular composition occurring between 7 and 28 days would require protein turnover and thus asked whether the 30 synapse subtypes with different rates of PSD95 turnover were differentially impacted by injury. To examine the relationship between the temporal evolution of the injury response and changes in the density of subtypes with different rates of PSD95 turnover,^7^ we first ranked the 30 subtypes according to the significance of their change (increase or decrease) (*p* < 0.05, Bayesian estimation with Benjamini-Hochberg correction) and marked the top six SPL (6, 8, 11, 28, 29, and 31) and top six LPL (2, 3, 5, 20, 34, and 35) subtypes (Figure 3A; Data S11, S12, S13, and S14). Next, heatmaps were plotted, ranked according to protein lifetime (Figure 3B; Data S11, S12, S13, and S14). These plots show several striking features. As shown in the heatmaps in Figure 3B, at 28 days, there is a blue vertical stripe (boxed in red) encompassing the leftmost ten subtypes in both ipsilateral and contralateral hemispheres, indicating that by 28 days there is widespread reduction in LPL synapses. Correspondingly, there is a general increase in the number of SPL synapse subtypes. Moreover, this pattern is not present at 7 days, indicating that it evolves after the first week following injury. Indeed, at 7 days, there is a reduced density of SPL synapses, particularly within the ipsilateral isocortex, indicating that these synapse subtypes underpin the synapse density loss described earlier. Collectively, this analysis highlights an initial decrease in SPL synapses and an increase in LPL synapses at 7 days and the reversal of this relationship by 28 days. These results indicate that synapses with different rates of PSD95 turnover respond differentially to TBI.

**Figure 3:**
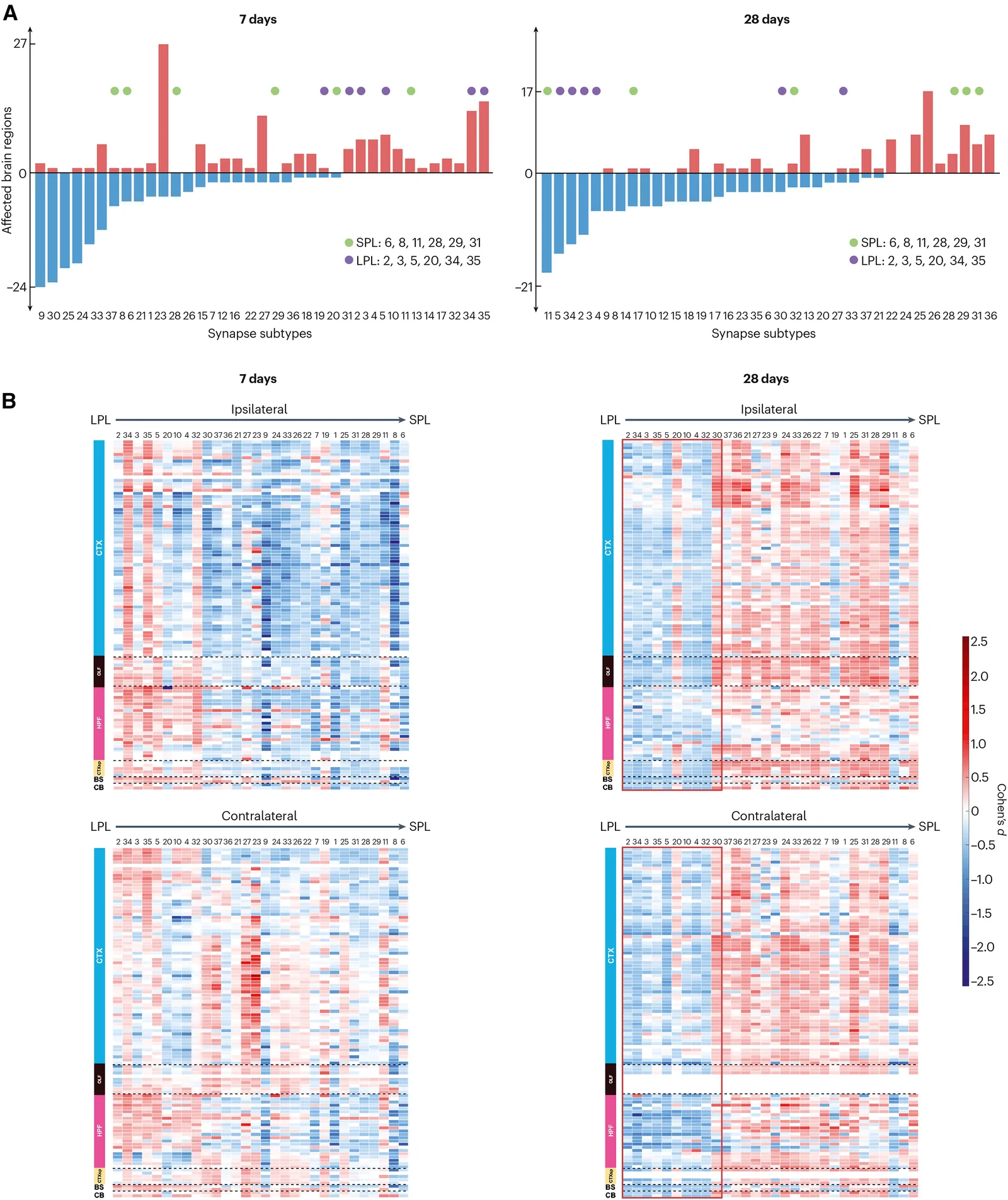
LFPI differentially impacts synapse subtypes with different protein lifetimes (A) Bars show the number of brain subregions in which the density of that subtype was significantly increased (red, upward) or decreased (blue, downward) in LFPI compared with sham-LFPI mice at 7 days (left) and 28 days (right) post-injury (*p* < 0.05, Bayesian estimation with Benjamini-Hochberg correction). Within each image, subtypes are ranked independently by the number of subregions in which that subtype was significantly reduced (leftmost) to increased (rightmost). The colored dots label 6 SPL subtypes with the shortest protein lifetime (green) and 6 LPL subtypes with the longest protein lifetime (purple). LPL subtypes cluster toward the right (fewest subregions reduced) at 7 days and shift toward the left (most subregions reduced) by 28 days, with SPL subtypes showing the opposite movement, illustrating the temporal reversal in subtype-selective vulnerability. (B) Heatmaps showing differences (Cohen’s *d*, key on right) in the synapse subtype density between LFPI and sham-LFPI groups. Subtypes are ranked from left to right according to PSD95 protein lifetime (LPL, longest protein lifetime on left; and SPL, shortest protein lifetime on right) across 111 brain subregions (both ipsilateral and contralateral) across two time points (7 and 28 days). LPL synapses are boxed in red in 28-day samples.

### Microglia show region- and time-dependent responses to brain injury

TBI is known to induce reactive responses in microglia that may contribute to synaptic damage.^4^^,^^23^^,^^24^^,^^25^ We therefore examined the spatiotemporal distribution of microglia by staining serial brain sections for the microglial marker IBA1 (AIF1) (Figure S2). Injury induced a marked increase in the IBA1 area in the ipsilateral hemisphere, and by 28 days, there was an additional increase in the contralateral hemisphere (Figure 4A; Data S16). A similar but less pronounced response was observed after sham-LFPI. Examination of the cortex, hippocampus, thalamus, and hypothalamus showed that IBA1 increases were predominant in the cortex and hippocampus (Figure 4B; Data S16). Interestingly, at 28 days when the cortex and hippocampus showed the maximal IBA1 area increase, there was an overall decrease in the thalamus and hypothalamus, potentially reflecting the redistribution or migration of microglia toward cortical and hippocampal regions.

**Figure 4:**
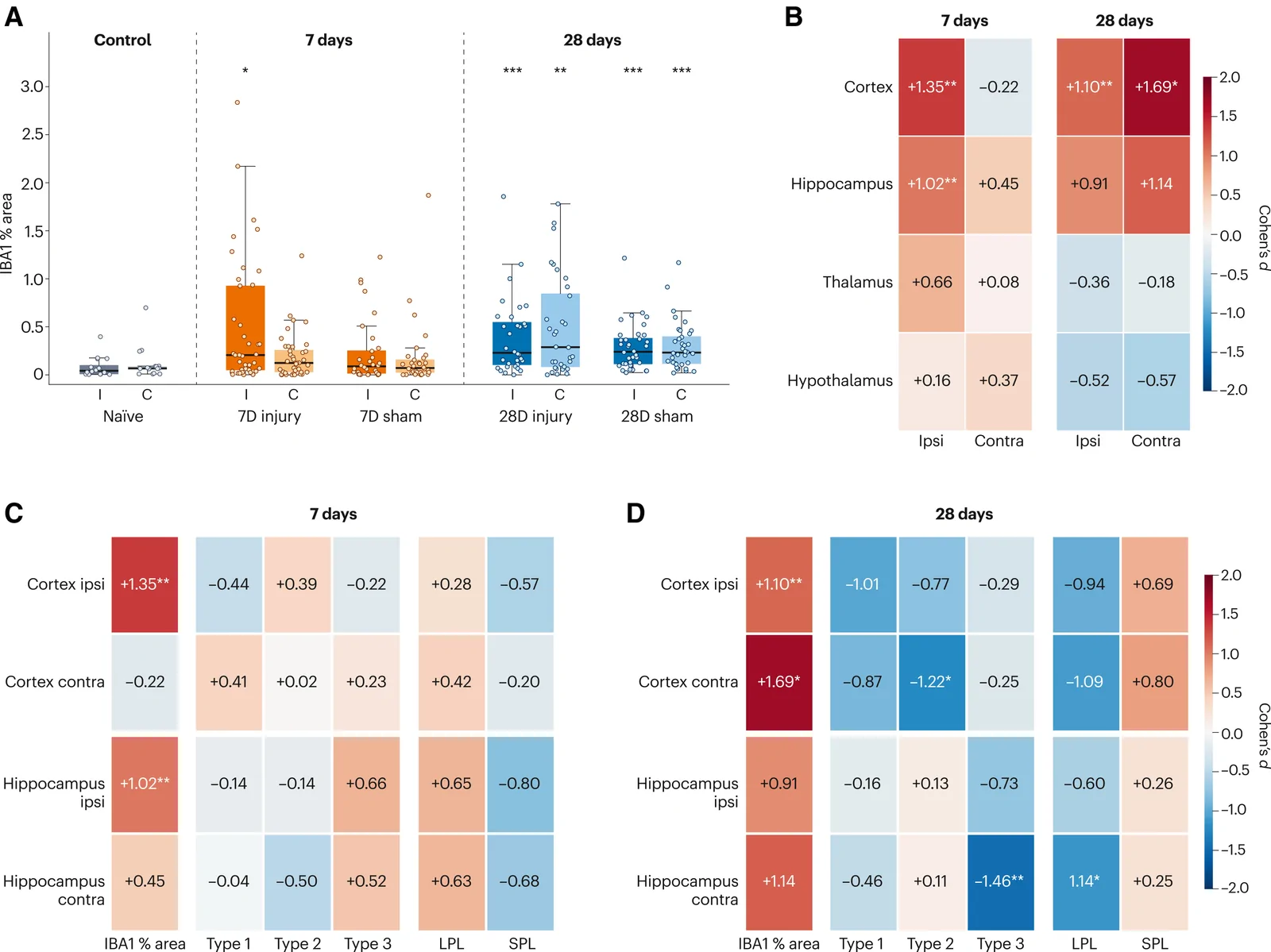
Relationship between microglia and synapse type responses to brain injury (A) The area (%) of the ipsilateral (I) and contralateral (C) hemispheres stained with IBA1 in naive, injury, and sham mice at 7 and 28 days. Boxplots show the median (center line) and interquartile range (box limits); whiskers extend to 1.5× the interquartile range, and each point represents one brain region per mouse. Asterisks indicate comparison with the corresponding naive hemisphere (Mann-Whitney U test). (B) The change (Cohen’s *d*) in IBA1 staining in brain regions comparing LFPI with sham-LFPI (statistical significance tested with Mann-Whitney U test). (C and D) Pearson correlation between change in IBA1 area and synapse type (1–3 and LPL, SPL) at 7 days (C) and 28 days (D). ∗*p* < 0.05; ∗∗*p* < 0.01; ∗∗∗*p* < 0.001.

We then asked whether microglial changes were related to alterations in type 1, 2, or 3 synapse density (Figures 4C and 4D; Data S16). At 28 days, the increases in IBA1 staining in the cortex were associated with a reduction in density in all three synapse types, in contrast to 7 days, which showed smaller changes. Similarly, in the hippocampus, the increase in IBA1 staining at 28 days was associated with a reduction in type 1 and 3 synapses, with less clear trends at 7 days. Next, we asked if the IBA1 changes correlated with changes in the SPL and LPL synapses (Figures 4C and 4D; Data S16). At day 28, the increase in IBA1 labeling correlated with increases in SPL synapses and decreases in LPL synapses. At 7 days, this relationship was reversed.

## Discussion

This study reveals that a single TBI event induces progressive and distributed reorganization of synaptome architecture rather than uniform synapse depletion. Although total whole-brain synapse number was preserved, specific synapse types and subtypes exhibited marked region- and time-dependent vulnerability. These findings demonstrate that TBI primarily disrupts the molecular composition and balance of synapse populations across the brain, redefining synaptic pathology after injury as architectural reprogramming rather than simple synapse loss.

A striking feature of the injury response was a temporally structured, two-phase rebalancing of synapses defined by protein lifetime. Early after injury, synapses with short PSD95 protein lifetimes were preferentially reduced, accompanied by a relative expansion of LPL synapses. By 28 days, this relationship reversed, with widespread reduction of LPL synapses and compensatory increases in SPL subtypes. This bidirectional shift, which could not be simply explained by increased IBA1 labeling, suggests that proteostasis is a key determinant of synaptic resilience and remodeling in TBI. SPL synapses may be acutely vulnerable to mechanical or inflammatory perturbation, whereas delayed reorganization leads to their recovery and loss of LPL synapses. Future studies measuring the rate of protein turnover using HaloTag mice^7^ could potentially identify the role of cytokines and other factors in synaptic proteostasis.

A central question raised by these findings is the extent to which microglia actively drive synaptome remodeling. Microglia are established mediators of activity- and injury-dependent synapse elimination, engulfing pre- and postsynaptic elements and tagging synapses for removal through complement-dependent and complement-independent mechanisms.^23^^,^^24^^,^^25^ In our data, the regional and temporal coupling between IBA1 area and synapse-type change—in particular the association at 28 days between increased cortical and hippocampal IBA1 labeling, loss of LPL synapses, and recovery of SPL synapses—is consistent with a model in which microglial activity participates in the subtype-selective, proteostasis-linked rebalancing of the synaptome. The reversal of this relationship between 7 and 28 days further suggests that any microglial contribution is temporally staged. These associations are, however, correlational: our region-level measurements cannot establish that microglia causally mediate the gain or loss of synapse subtypes. These interpretations are further constrained by the limitations of an IBA1-based approach. IBA1 labels microglia irrespective of their functional state and provides only a regional and morphological proxy for microglial activity.^26^ It does not distinguish homeostatic from activated, phagocytic, or pruning phenotypes nor does it report direct microglia-synapse contact or engulfment of specific synapse types. Establishing whether microglia causally drive this subtype-selective remodeling will require moving beyond correlative IBA1-area measurements to interventional approaches—including timed microglial depletion or complement-pathway manipulation to test necessity, alongside synaptic-engulfment assays that quantify internalized PSD95-eGFP and SAP102-mKO2 material within state-defined microglia. Combining such manipulations with PSD95-HaloTag measurements of protein lifetime would further test the specific prediction that microglia preferentially remove LPL synapses, directly linking microglial activity to the proteostasis-dependent rebalancing of the synaptome.

The injury-induced rebalancing of the synaptome mirrors patterns observed in genetic mutation, aging, sleep deprivation, and experience-dependent plasticity.^7^^,^^9^^,^^11^^,^^12^ Across these diverse conditions, excitatory synapse populations undergo selective type and subtype vulnerability and large-scale architectural reorganization. This conservation suggests that synaptome remodeling represents a general systems-level mechanism for adapting—or maladapting—to perturbation. Consistent with this model, increasing age is a well-established risk factor for poor outcome,^27^^,^^28^ environmental enrichment promotes rehabilitation,^29^ and sleep disturbances adversely affect the clinical course of TBI recovery.^30^^,^^31^ Our study also provides insights into how the cognitive and behavioral impairments arise from TBI, since the synaptome architecture is a mechanism for encoding innate and learned behavioral representations,^7^^,^^10^^,^^12^^,^^32^^,^^33^ and its disruption and reorganization would be anticipated to result in altered cognition and behavioral phenotypes.

The distributed nature of synaptome remodeling indicates that injury effects are not confined to the impact site but propagate across interconnected regions, including the contralateral hemisphere. The observation that craniotomy alone induces measurable synaptome alterations underscores the sensitivity of synapse populations to surgical and inflammatory perturbations and has important implications for experimental paradigms involving cranial windows and for interpretation of injury models. The triggering of synaptome changes by craniotomy indicates that studies that image synapse protein or dendritic spine dynamics may be overestimating the dynamics of normal synapses.

The synaptome changes in the craniotomy-only (sham-LFPI) group have implications for the interpretation of our LFPI and sham-LFPI comparison. As both groups underwent identical craniotomies, this comparison aims to isolate the impact of the fluid percussion pulse as a mechanism of injury. We additionally analyzed LFPI against surgery-naive mice, which captures the combined contribution of craniotomy and percussion injury and revealed a convergent pattern of synaptome architecture disruption. Nonetheless, we cannot exclude the possibility of non-additive interactions between craniotomy and fluid percussion injury at specific subtypes or brain regions. Therefore, the LFPI-induced changes reported here should be interpreted as effects over and above a craniotomized baseline rather than against an uninjured brain.

Our findings indicate that widespread changes in the molecular composition of excitatory synapses occur in response to a mild TBI induced by craniotomy and to a more severe impact. The spatial and temporal reorganization of the synaptome may underlie the altered cognitive and behavioral features associated with TBI. It will be of interest to further explore the mechanisms of synapse type and subtype-selective vulnerability. Such studies could include investigation of the role of microglia activation states. It will also be important to ask whether the rate of protein turnover is modified as the TBI response evolves post-injury, which could be assessed with PSD95-HaloTag mice.^7^ Synaptome mapping provides a powerful approach to understanding synapse vulnerability and resilience to brain injuries and could be a useful tool for testing the efficacy of therapeutic interventions.

### Limitations of the study

Our study has several limitations. First, synaptome mapping was performed on a single mid-coronal plane (bregma −1.9 mm), which, although capturing a range of cortical, hippocampal, and thalamic regions, does not represent the full rostrocaudal extent of the brain. Second, our design was restricted to two time points (7 and 28 days). We are therefore unable to resolve the acute synaptic response in the first week post-injury or determine whether the contralateral synapse loss observed at 28 days progresses, stabilizes, or recovers at later time points. Coupled to this, we did not assess behavioral or cognitive outcomes and therefore are unable to establish the functional consequences of the synaptome remodeling described. Finally, our IBA1-based analysis provides a correlative, regional measure of microglial activity and cannot establish a causal role for microglia in subtype-selective synapse loss.

## Resource availability

### Lead contact

Further information and requests for resources and reagents should be directed to and will be fulfilled by the lead contact, Seth G.N. Grant (seth.grant@ed.ac.uk).

### Materials availability

The PSD95-eGFP and SAP102-mKO2 knockin mouse lines used in this study were generated previously^10^ and are available from the lead contact under a completed materials transfer agreement. This study did not generate new unique reagents.

### Data and code availability

• The synapse TBI data have been deposited at the Open Science Framework and are publicly available as of the date of publication. Accession numbers are listed in the key resources table.
• All original code (the SYNMAP synaptome-mapping pipeline) is publicly available as of the date of publication at the GitLab repository (https://git.ecdf.ed.ac.uk/zqiu/mouse_synaptome_mapping_fullsample) under a CC-BY license.
• Any additional information required to reanalyze the data reported in this paper is available from the lead contact upon request.

## Acknowledgments

We extend our appreciation to K. Elsegood and C. McLaughlin for laboratory support, D. Dominic for assistance in data management, C. Davey for editing, and D. Maizels for artwork. Funding was provided by a Wellcome Trust Translational Medicine and Therapeutics grant (106851/Z/15/Z) to A.A.B.J.

## Author contributions

Conceptualization, A.A.B.J. and S.G.N.G.; methodology, A.A.B.J., Z.Q., J.R., and S.G.N.G.; investigation, A.A.B.J., R.G., Z.Q., and J.R.; writing – original draft, A.A.B.J. and S.G.N.G.; writing – review and editing, A.A.B.J., Z.Q., R.G., J.R., and S.G.N.G.; funding acquisition, A.A.B.J. and S.G.N.G.; resources, J.R., Z.Q., and S.G.N.G.; supervision, J.R. and S.G.N.G.

## Declaration of interests

The authors declare no competing interests.

## STAR★Methods

### Key resources table

**Table:** 

REAGENT or RESOURCE	SOURCE	IDENTIFIER
**Antibodies**		

Goat anti-Iba1 polyclonal IgG	Abcam	Cat# ab5076; RRID: AB_2224402
Donkey anti-goat IgG H&L, Alexa Fluor 647	Abcam	Cat# ab150131; RRID: AB_2732857
Rabbit anti-GFAP polyclonal	Abcam	Cat# ab7260; RRID: AB_305808
Donkey anti-rabbit Alexa Fluor 546	Abcam	Cat# ab10040; RRID: AB_2534016
Mouse anti-amyloid precursor protein (clone 22C11)	Millipore	Cat# MAB348; RRID: AB_2056583

**Chemicals, peptides, and recombinant proteins**		

Isoflurane	Merial	N/A
Phenobarbital sodium 20% (Euthatal)	Merial Animal Health	N/A
Paraformaldehyde, 16%	Alfa Aesar	N/A
Phosphate-buffered saline (PBS)	Fisher Scientific	N/A
Sucrose	VWR Chemicals	N/A
Optimal cutting temperature compound (OCT)	VWR International	N/A
Isopentane (2-methylbutane)	Sigma-Aldrich	N/A
Bovine serum albumin (BSA)	Sigma-Aldrich	N/A
Triton X-100	Sigma-Aldrich	N/A
DAPI	Sigma-Aldrich	Cat# D9542
DABCO (1,4-diazabicyclo[2.2.2]octane)	Sigma-Aldrich	Cat# D27802
Shandon Harris Haematoxylin, acidified	Thermo Fisher Scientific	Cat# 6765003
Shandon Eosin Y, alcoholic	Thermo Fisher Scientific	Cat# 6766007
DPX mountant	Sigma-Aldrich	Cat# 06522
Mowiol 4–88	Calbiochem/Sigma-Aldrich	N/A
Bupivacaine	AstraZeneca	N/A
3M Vetbond tissue adhesive	3M	N/A
Loctite Power Flex Gel	Loctite (Henkel)	N/A
Dental cement (Simplex Red)	Associated Dental Products	N/A

**Critical commercial assays**		

Novolink Max Polymer Detection System	Leica Biosystems	Cat# RE7280-CE

**Deposited data**		

TBI Synaptome source data (16 Excel files)	This paper	https://doi.org/10.17605/OSF.IO/EYD2J

**Experimental models: Organisms/strains**		

Mouse: PSD95-eGFP knock-in (*Psd95*^eGFP/eGFP^; C57BL/6J)	Zhu et al.^10^	N/A
Mouse: SAP102-mKO2 knock-in (*Sap102*^mKO2/y^; C57BL/6J)	Zhu et al.^10^	N/A
Mouse: wild-type C57BL/6	Charles River Laboratories	N/A

**Software and algorithms**		

SYNMAP synaptome-mapping pipeline	Zhu et al.^10^	https://git.ecdf.ed.ac.uk/zqiu/mouse_synaptome_mapping_fullsample
GraphPad Prism 7	GraphPad Software	RRID:SCR_002798
ImageJ	NIH	RRID:SCR_003070
ZEN	Carl Zeiss	RRID:SCR_013672
Andor iQ acquisition software	Andor Technology	N/A
Random sequence generator	random.org	https://www.random.org
MATLAB	MathWorks	RRID:SCR_001622
Python	Python Software Foundation	RRID:SCR_008394

**Other**		

Vicryl 5–0 suture	Ethicon	N/A
Fluid percussion injury device	Custom Design and Fabrication, Virginia Commonwealth University	N/A
Stereotaxic frame, Model 900	David Kopf Instruments	N/A
Homoeothermic Blanket Control Unit	Harvard Apparatus	N/A
NX70 cryostat	Thermo Scientific	N/A
Superfrost Plus glass slides	Thermo Scientific	N/A
Andor Revolution XDi spinning disk confocal (CSU-W1)	Andor Technology	N/A
Andor iXon Ultra back-illuminated EMCCD camera	Andor Technology	N/A
UPlanSAPO 100× oil-immersion objective (NA 1.4)	Olympus	N/A

### Experimental model and study participant details

#### Animals

Animal experimentation was undertaken in accordance with Home Office guidelines [Animal (Scientific Procedures) Act 1986] and was approved by The University of Edinburgh. PSD95-eGFP and SAP102-mKO2 knock-in mice were used.^10^ Males homozygous for PSD95-eGFP and hemizygous for SAP102-mKO2 and aged 8-16 weeks were included in the experiments. The mice were allowed to acclimatize in the animal unit for at least 7 days prior to experimentation. The mice were kept in a 12-hour light/dark cycle and given free access to food and water before and after the experimental procedure. The PSD95-eGFP and SAP102-mKO2 lines were maintained on a C57BL/6J background; mice used in the injury experiments were male, homozygous for PSD95-eGFP and hemizygous for SAP102-mKO2, and were experimentally naïve and in good health at the time of surgery. Surgical procedures were performed under UK Home Office Project Licence 70/7984 under establishment licence (PEL) 60/2605, in accordance with the Animals (Scientific Procedures) Act 1986, and were approved by the University of Edinburgh Animal Welfare and Ethical Review Body. A separate cohort of adult male wild-type C57BL/6 mice (Charles River Laboratories; 8–12 weeks of age) was used to characterize the LFPI model.

### Method details

#### Animals per group

Synaptome mapping was performed on *n* = 11 mice at 7 days post-injury (LFPI) and *n* = 8 sham-LFPI mice at 7 days; *n* = 10 LFPI and *n* = 10 sham-LFPI mice at 28 days; and *n* = 5 surgery-naïve mice. A separate cohort of *n* = 26 wild-type C57BL/6 mice (*n* = 15 LFPI, *n* = 11 sham-LFPI) was used to characterise the injury model (Figure S1). Sample size was determined *a priori* as described in experimental design. Twenty animals were excluded according to the pre-specified criteria (dural tear during craniectomy, dural rupture following the fluid-percussion pulse, or post-injury paralysis lasting > 30 min); exclusions were made before imaging and before unblinding. Group sizes are restated in each figure legend.

#### Lateral fluid percussion injury model

Mice were anaesthetized with 5% isoflurane (Merial, UK) in 100% O_2_ for 2 minutes prior to surgery. The mice were then weighed and placed in a stereotaxic frame (Model 900, David Kopf Instruments). A nose cone was used to maintain anaesthesia. During surgery, mouse body temperature was maintained at 37±0.5°C using a heat pad and rectal thermometer probe (Homoeothermic Blanket Control Unit, Harvard Apparatus). A razor was then used to shave a small patch of hair from the top of the mouse’s head and eye ointment (Xailin) was applied to the eyes. A midline scalp incision was fashioned and the skin flaps reflected using skin clips. A plastic guide was glued onto the skull using 3M Vetbond and a lateral 2 mm circular craniectomy was then trephined (Miltex) midway between the bregma and lambda. Care was taken not to injure underlying dura. An injury hub was created by cutting off the female end of a Luer-Loc needle hub. This was secured over the craniectomy site using glue (Loctite Power Flex Gel) and dental cement (Simplex Red, Associated Dental Products). The mice were then removed from the stereotactic frame and the depth of anaesthesia reduced until they responded to tail pinch. The mice were then placed in the anaesthetic box for 1 minute. Once complete, the injury hub was filled with normal saline and attached to the fluid percussion device (Custom Design and Fabrication, Virginia Commonwealth University). When they showed response to tail stimulus (to standardize anaesthesia levels), a mild to moderate injury (1.1-1.5 atm) was inflicted. This was determined by oscilloscope attached to the fluid percussion device. Sham mice had the exact same procedure except the release of the pendulum to inflict the percussion injury was omitted. Following this, the mice were disconnected from the fluid percussion device and placed on their back to measure their righting time – a marker of the neurological severity of the injury. Exclusion criteria were defined as follows: dural tear during craniectomy; dural rupture following application of fluid percussion or post-injury paralysis lasting more than 30 minutes. Following righting, the mice were then re-anesthetized and had the hub removed and the skin incision sutured using 5-0 Vicryl (Ethicon). A 2 mg/kg dose of Bupivacaine (AstraZeneca) was applied to the wound to manage pain. Mice were then placed in a heated recovery chamber at 30°C (Lyon Electric Company) for 30 minutes prior to being returned to a cage. Mice were followed up to 7 days (7D) or 28 days (28D). Mice were initially monitored daily for the 5 days postoperatively. Thereafter, they were assessed once a week until the end of the experiment. Prior to the synaptome experiment, the LFPI model was comprehensively characterised in a cohort of wild-type mice. Mice were randomly allocated to one of four cohorts (7-day injury, 7-day sham, 28-day injury and 28-day sham): as each batch became eligible, animals were and assigned to cohorts in a fixed order using a random-number sequence generated at random.org.

#### Tissue collection and processing

Mice were administered a terminal dose of 0.1 ml 20% phenobarbital sodium (Euthatal, Merial Animal Health) by intraperitoneal injection. Once the mice were fully anaesthetized (not responding to tail pinch), they were pinned to a cork board and underwent a thoracotomy. The heart was fully exposed and a small knick made in the right atrium. Subsequently, 10 ml phosphate-buffered saline (PBS, Fisher Scientific) was injected slowly into the left ventricle. This was followed by 10 ml 4% paraformaldehyde (PFA, prepared by diluting 16% Alfa Aesar 1:4 in PBS). The mice were then decapitated and the brain dissected free from the skull. It was post-fixed in 5 ml 4% PFA for 3-4 hours and then placed in a 30% sucrose solution (prepared by diluting sucrose (VWR Chemicals) in PBS) for 48 hours. The brain was then placed in a solution of 50:50 30% sucrose and Optimum Cutting Temperature medium (OCT, VWR International) for 1 hour before being embedded in a mould (Sigma-Aldrich) in OCT. This was achieved by immersing the moulds in a beaker of isopentane (Sigma-Aldrich) which was then placed in liquid nitrogen. Once frozen, the brains were kept at -80°C until sectioned. An NX70 Cryostat (Thermo Scientific) was used to cut coronal sections 18 microns in depth. A small drop of PBS was placed on a Superfrost Plus glass slide (Thermo Scientific) before sections were collected. Slides were left to dry at 4°C overnight, then stored at -80°C until further analysis. Sections were kept away from light to minimize loss of fluorescence. Following fixation, brains were coded by an independent member of the laboratory who maintained a central register that was inaccessible to the primary investigator; all subsequent image acquisition and analysis were performed blinded to experimental group, and the code was broken only after the raw sub-regional puncta data had been generated.

#### Haematoxylin and Eosin (H+E) staining

Selected slides were washed in a 1% PBS bath for 5 minutes. After this, slides were hydrated through graduated ethanol concentrations followed by rinsing in running water for 5 minutes. The slides were then placed in filtered Shandon ready-made Harris Haematoxylin Acidified (ThermoFisher Scientific #6765003) for 3 minutes followed by rinsing in running water. The haematoxylin was then differentiated in 1% acid alcohol for 10 seconds. After rinsing, the slides were then placed in Scott’s Tap Water substitute (1 litre distilled water, 20g MgSO_4_, 3.5g NaHCO_3_) for 2 minutes. From there, the slides were placed in Shandon EosinY Alcoholic (ThermoFisher Scientific #6766007). Following rinsing in running water, the slides were dehydrated through increasing graduated alcohol concentrations. The slides were finally cover-slipped using DPX (Sigma #06522). All stages were performed at room temperature.

#### Astrocyte and axonal staining

In the wild-type LFPI characterization cohort, astrocytes were labelled by fluorescence immunohistochemistry: sections were washed of OCT in 1% PBS, blocked in 5% BSA/0.2% Triton X-100, and incubated overnight at 4 °C with rabbit anti-GFAP (Abcam ab7260; 1:500). Bound antibody was detected with donkey anti-rabbit Alexa Fluor 546 (Abcam ab10040; 1:300), nuclei were counterstained with DAPI, and sections were mounted in Mowiol. GFAP images were acquired on a Zeiss Axioscan.Z1 (20×, NA 0.8), and GFAP-positive cells were quantified within delineated regions of interest in ImageJ by intensity thresholding and semi-automated particle analysis, blinded to group. Traumatic axonal injury was assessed by chromogenic immunohistochemistry for amyloid precursor protein (APP): after ethanol rehydration and a peroxidase block, sections underwent heat-mediated antigen retrieval in citrate buffer (pH 6) and were incubated with mouse anti-APP A4 (clone 22C11; Millipore MAB348; 1:1000) using the Leica NovoLink Max Polymer detection system, developed with DAB, counterstained with haematoxylin, and mounted in DPX. APP-positive axonal varicosities (dark aggregates) in the ipsilateral and contralateral corpus callosum were counted by semi-quantitative light microscopy (Olympus BX-51, 20×), blinded to group.

#### Microglial imaging and quantification

Sections were washed of OCT in 1% PBS for 5 minutes. A 10% BSA blocking solution was applied for 1 hour. The primary antibody goat IBA1 polyclonal IgG (Abcam ab5076) was applied at 1:150 and incubated at room temperature for 90 minutes. Sections were then washed with Tris-buffered saline pH 7.0 (TBS). The secondary antibody donkey anti-goat Alexa Fluor 647 IgG H&L (Abcam ab150131) was applied at 1:500 for 1 hour at room temperature, followed by three 5-minute TBS washes. Immunofluorescence imaging was performed using a Zeiss Axioscan.Z1 at 20× using a Zeiss Plan-Apochromat lens (N.A. 0.8). Representative images are shown in Figure S2. The acquired CZI-format images were converted to 16-bit TIFFs using ZEN software (Zeiss) and the IBA1 channel was extracted. Anatomical regions of interest (cortex, hippocampus, thalamus, hypothalamus) were delineated for both the ipsilateral and contralateral hemispheres in ImageJ (Fiji) using the polygon selection tool, guided by the Allen Mouse Brain Atlas. Each IBA1 image was converted to 8-bit and a fixed intensity threshold (range 0–17 on a 0–255 scale) was applied identically across all sections, generating a binary image in which pixels above threshold corresponded to IBA1-positive staining and pixels below threshold to background. For each region of interest, the IBA1-labelled area was defined as the total pixel area above threshold within that region, expressed as a percentage of the total area of the region of interest. All images were analysed blinded to experimental group.

#### Spinning disc confocal microscopy

Image acquisition was performed using the Andor Revolution XDi system with Dual Nipkow disc (CSU-W1), which combines two pinholes of 25 and 50 μm. We utilized two laser wavelengths: 488 nm (eGFP) and 561 nm (mKO2). Light from the lasers was directed onto the sample using a dichroic mirror and quad filter. A 2x post-magnification lens was placed in front of the camera to increase sampling rate. The system also contained the Borealis Perfect Illumination Delivery which improves the uniformity of the field illumination. Images were acquired using an Olympus UplanSAPO 100x oil-immersion lens (N.A. 1.4) and detection was achieved using the Andor iXon Ultra monochrome back-illuminated EMCCD. Acquired images were 512 x 512 pixels and 16-bit depth. For whole-brain acquisition, images underwent mosaic tilling across a grid defined by the user. Within this grid, focus points were placed by the user which allowed for adaptive focusing throughout the imaged sample. No z-stacking was taken in images. Representative images of synaptic puncta are shown in Figure S1A. The final pixel dimension in the sample plane was 84 × 84 nm. Acquisition was controlled using the Andor iQ software.

#### Synaptome mapping pipeline

The synaptome mapping (SYNMAP) pipeline^8^^,^^10^ was developed and standardized to systematically map individual PSD95-eGFP and SAP102-mKO2 puncta across the entire brain. Using in-house developed deep-learning methods,^34^^,^^35^ SYNMAP comprises a sequence of automated image-analysis procedures that includes puncta detection, colocalization, classification and map reconstruction. To define the anatomical regions, manual delineation was employed using the Allen Mouse Brain Atlas as a guiding resource. For each punctum, intensity, size and morphological features were extracted at single-synapse resolution. These features were then used as inputs to an in-house unsupervised model that generated a hierarchical synapse catalogue comprising three primary classes (PSD95 only, SAP102 only, colocalized) and 37 subtypes. In our previous study,^7^ we quantified protein lifetime across 30 synapse subtypes that express PSD95 by tracking the temporal persistence of labelled PSD95 molecules. The six synapse subtypes with the longest turnover times were classified as long protein lifetime (LPL), and the six with the shortest turnover times were classified as short protein lifetime (SPL). Puncta detection and segmentation used a supervised machine-learning (Ensemble) classifier implemented in MATLAB and Python; a ground-truth training set was created by manually counting PSD95 and SAP102 puncta in randomly selected image subsets using the Cell Counter plugin in ImageJ (Fiji). For each detected punctum a region of interest was generated and three parameters were computed: density (mean number of puncta per tile), intensity (mean grey value of pixels within the punctum) and area (number of pixels within the punctum, converted to μm^2^ using a pixel area of 0.007056 μm^2^). For each brain, 222 sub-regions were manually delineated on a whole-brain montage using the polygon tool in ImageJ, guided by the Allen Mouse Brain Atlas, and the delineations were applied as binary masks to compute the median value of each parameter per sub-region.

#### Experimental design

The study compared LFPI (injury) and sham-LFPI (craniotomy only) mice at 7 and 28 days post-injury. A separate non-randomised surgery-naïve group was included as a control. Sample size was estimated from a pilot cohort of PSD95-eGFP SAP102-mKO2 mice followed to 7 days: an *a priori* power calculation based on ipsilateral-cortex PSD95 puncta density (α = 0.05, power = 0.95) indicated that nine mice per cohort were required. Allowing for an anticipated attrition of ∼30%, a minimum of 47 mice were randomized across the four cohorts. Animals were randomly allocated to cohorts (random.org) and all image acquisition and analysis were performed blinded to group. Pre-specified exclusion criteria were: dural tear during craniectomy; dural rupture following the fluid-percussion pulse; or post-injury paralysis lasting more than 30 minutes.

#### Data files descriptive titles

Data S1. Regional PSD95-eGFP puncta count, size, intensity and colocalisation in LFPI mice at 7 days, related to Figure 2 (comparison with LFPI-sham mice), S4 (comparison with surgery-naïve mice) and S5 (comparison with LFPI-sham mice).

Data S2. Regional PSD95-eGFP puncta count, size, intensity and colocalisation in sham-LFPI mice at 7 days, related to Figure 2 (comparison with LFPI mice) and S5 (comparison with LFPI mice).

Data S3. Regional PSD95-eGFP puncta count, size, intensity and colocalisation in LFPI mice at 28 days, related to Figure 2 (comparison with LFPI-sham mice), S4 (comparison with surgery-naïve mice) and S5 (comparison with LFPI-sham mice).

Data S4. Regional PSD95-eGFP puncta count, size, intensity and colocalisation in sham-LFPI mice at 28 days, related to Figure 2 (comparison with LFPI mice) and S5 (comparison with LFPI mice).

Data S5. Regional PSD95-eGFP puncta count, size, intensity and colocalisation in surgery-naïve mice, related to Figure S4 (comparison with LFPI mice).

Data S6. Regional SAP102-mKO2 puncta count, size, intensity and colocalisation in LFPI mice at 7 days, related to Figures (comparison with LFPI-sham mice), Figure S4 (comparison with surgery-naïve mice) and Figure S5 (comparison with LFPI-sham mice).

Data S7. Regional SAP102-mKO2 puncta count, size, intensity and colocalisation in sham-LFPI mice at 7 days, related to Figure 2 (comparison with LFPI mice) and Figure S5 (comparison with LFPI mice).

Data S8. Regional SAP102-mKO2 puncta count, size, intensity and colocalisation in LFPI mice at 28 days, related to Figures (comparison with LFPI-sham mice), Figure S4 (comparison with surgery-naïve mice) and Figure S5 (comparison with LFPI-sham mice).

Data S9. Regional SAP102-mKO2 puncta count, size, intensity and colocalisation in sham-LFPI mice at 28 days, related to Figure 2 (comparison with LFPI mice) and Figure S5 (comparison with LFPI mice).

Data S10. Regional SAP102-mKO2 puncta count, size, intensity and colocalisation in surgery-naïve mice, related to Figure S4 (comparison with LFPI mice).

Data S11. Regional densities of 37 excitatory synapse subtypes in LFPI mice at 7 days, related to Figure 1 (total number of subtype puncta comparison with sham-LFPI and surgery-naïve), 3 (comparison with sham-LFPI) and Figure S6 (comparison with sham-LFPI).

Data S12. Regional densities of 37 excitatory synapse subtypes in sham-LFPI mice at 7 days, related to Figure 1 (total number of subtype puncta comparison with LFPI and surgery-naïve), 3 (comparison with LFPI) and Figure S6 (comparison with LFPI).

Data S13. Regional densities of 37 excitatory synapse subtypes in LFPI mice at 28 days, related to Figure 1 (total number of subtype puncta comparison with sham-LFPI and surgery-naïve), 3 (comparison with sham-LFPI) and Figure S6 (comparison with sham-LFPI).

Data S14. Regional densities of 37 excitatory synapse subtypes in sham-LFPI mice at 28 days, related to Figure 1 (total number of subtype puncta comparison with LFPI and surgery-naïve), 3 (comparison with LFPI) and Figure S6 (comparison with LFPI).

Data S15. Regional densities of 37 excitatory synapse subtypes in surgery-naïve mice, related to Figure 1 (total number of subtype puncta comparison with sham-LFPI and surgery-naïve).

Data S16. Regional IBA1 microglial labelling alongside synapse density and subtype metrics across all cohorts, related to Figure 4.

### Quantification and statistical analysis

#### Statistical reporting

Full statistical details for every experiment are given in the corresponding figure legends (Figures 1, 2, 3, and 4; Figures S1–S6 and, where relevant, in the Results text. Unless stated otherwise, *n* refers to the number of mice; no animal contributed more than one brain section to any analysis, and no technical replicates were pooled as independent observations. For synaptome analyses, the unit of measurement within each animal was the anatomical subregion: 222 subregions were delineated per brain (111 in the ipsilateral and 111 in the contralateral hemisphere), and the value for each subregion in each animal was the median of the per-punctum measurements within that subregion (density, mean intensity or area), so that each animal contributed one value per subregion per parameter. Statistical significance was defined as *p* < 0.05 throughout (∗*p* < 0.05, ∗∗*p* < 0.01, ∗∗∗*p* < 0.001); non-significant comparisons are indicated as ns (*p* > 0.05). All tests were two-tailed. Investigators were blinded to experimental group during all image acquisition and analysis, and the group code was broken only after the raw sub-regional puncta data had been generated (see STAR Methods).

#### Software

Puncta detection, segmentation and classification were performed with the SYNMAP synaptome-mapping pipeline implemented in MATLAB (MathWorks; RRID:SCR_001622) and Python (Python Software Foundation; RRID:SCR_008394). Region-of-interest delineation and immunohistochemical quantification were performed in ImageJ/Fiji (NIH; RRID:SCR_003070). Bayesian estimation, Benjamini–Hochberg correction and Cohen's d computation were performed using custom scripts in MATLAB and Python. All other statistical tests and the generation of bar, box and correlation plots were performed in Python (Python Software Foundation; RRID:SCR_008394). Cohort allocation used the random-number sequence generator at random.org. Software versions and identifiers are listed in the key resources table.

#### Astroglial and amyloid quantification and analysis

Regional differences in IBA1 area between LFPI and sham-LFPI mice were tested with the Mann–Whitney U test (Figures 4A and 4B). The relationships between the change in IBA1 area and the change in synapse type (Types 1–3) or protein-lifetime class (SPL, LPL) density were assessed by two-tailed Pearson correlation (Figures 4C and 4D); r values with associated *p* values are reported in the figure. Correlation analyses are correlative and were not corrected for multiple comparisons; they are interpreted as descriptive. In the wild-type characterisation cohort (Figure S1), righting-reflex time and quantitative immunostaining (GFAP-positive cell counts) were compared between LFPI and sham-LFPI groups using the Mann–Whitney U test. Data are shown as median with IQR, with individual animals plotted. Categorical corpus-callosal APP findings (presence of APP-positive axonal varicosities) were compared using the χ^2^ test.

#### Whole-brain and regional puncta counts

Total synapse numbers were calculated by adding up number of synapse subtypes across subregions. Whole-brain synapse puncta counts (Figure 1B) and puncta counts in the ipsilateral cortex underlying the craniotomy (Figure 1C) were compared across cohorts by one-way ANOVA with Tukey's multiple comparisons test. Normality was assessed with the Shapiro–Wilk test prior to parametric testing. Data are displayed as box plots in which the centre line is the median, the box limits are the first and third quartiles, and the upper and lower whiskers indicate the 95th and 5th percentiles; individual points represent individual mice. The whole-brain puncta count reported in the Results is given as the median with interquartile range (IQR) and first and third quartiles.

#### Bayesian estimation of between-group differences in synaptome parameters

Between-group differences in synaptome parameters (PSD95 and SAP102 puncta density, mean intensity and size; the densities of the 3 synapse types and 37 synapse subtypes; and synapse diversity) were assessed subregion-by-subregion using Bayesian estimation.^36^ Because 222 subregions were tested for each parameter, *p* values were corrected for multiple comparisons across all subregions using the Benjamini–Hochberg false discovery rate procedure; subregions surviving correction at *p* < 0.05 are indicated in the relevant figures (Figures 2 and 3; Figures S4 and S5). Comparisons were made between LFPI and sham-LFPI, and between LFPI or sham-LFPI and the surgery-naïve group, at each time point separately. Time points were not compared directly, as the design is cross-sectional.

#### Effect sizes

The magnitude of between-group differences is reported throughout as the Cohen's *d*^37^ effect size and displayed as spatial heatmaps registered to the Allen Mouse Brain Atlas. Cohen's d was calculated as the difference between the group means divided by the pooled standard deviation:

(Equation 1) $$d=\frac{x_{1}^{\prime }-x_{2}^{\prime }}{S}$$

where *x* is the mean of each group and *s* is the pooled standard deviation. Positive *d* values (red in heatmaps) denote an increase and negative values (blue) a decrease in the LFPI group relative to the comparison group. In Figure 3A, bars represent the number of subregions in which a given subtype was significantly increased or decreased (*p* < 0.05, Bayesian estimation with Benjamini–Hochberg correction); here the unit counted is the subregion, not the animal.

## Supplemental information

Document S1. Figures S1–S6

Data S1. PSD95 puncta measurements in LFPI mice at 7 days, related to Figures 2, S4, and S5 Per-region PSD95-eGFP puncta count, mean punctum area, mean punctum intensity and proportion of puncta colocalised with SAP102-mKO2, in the ipsilateral and contralateral hemispheres at Bregma −1.9 mm. One worksheet per mouse (n = 11). File: s1_7d_injury_psd95.xlsx

Data S2. PSD95 puncta measurements in sham-LFPI mice at 7 days, related to Figures 2 and S5 Per-region PSD95-eGFP puncta count, mean punctum area, mean punctum intensity and proportion of puncta colocalised with SAP102-mKO2, in the ipsilateral and contralateral hemispheres at Bregma −1.9 mm. One worksheet per mouse (n = 8). File: s2_7d_sham_psd95.xlsx

Data S3. PSD95 puncta measurements in LFPI mice at 28 days, related to Figures 2, S4, and S5 Per-region PSD95-eGFP puncta count, mean punctum area, mean punctum intensity and proportion of puncta colocalised with SAP102-mKO2, in the ipsilateral and contralateral hemispheres at Bregma −1.9 mm. One worksheet per mouse (n = 10). File: s3_28d_injury_psd95.xlsx

Data S4. PSD95 puncta measurements in sham-LFPI mice at 28 days, related to Figures 2 and S5 Per-region PSD95-eGFP puncta count, mean punctum area, mean punctum intensity and proportion of puncta colocalised with SAP102-mKO2, in the ipsilateral and contralateral hemispheres at Bregma −1.9 mm. One worksheet per mouse (n = 10). File: s4_28d_sham_psd95.xlsx

Data S5. PSD95 puncta measurements in surgery-naive mice, related to Figure S4 Per-region PSD95-eGFP puncta count, mean punctum area, mean punctum intensity and proportion of puncta colocalised with SAP102-mKO2, in the ipsilateral and contralateral hemispheres at Bregma −1.9 mm. One worksheet per mouse (n = 5). File: s5_naive_psd95.xlsx

Data S6. SAP102 puncta measurements in LFPI mice at 7 days, related to Figures 2, S4, and S5 Per-region SAP102-mKO2 puncta count, mean punctum area, mean punctum intensity and proportion of puncta colocalised with PSD95-eGFP, in the ipsilateral and contralateral hemispheres at Bregma −1.9 mm. One worksheet per mouse (n = 11). File: s6_7d_injury_sap102.xlsx

Data S7. SAP102 puncta measurements in sham-LFPI mice at 7 days, related to Figures 2 and S5 Per-region SAP102-mKO2 puncta count, mean punctum area, mean punctum intensity and proportion of puncta colocalised with PSD95-eGFP, in the ipsilateral and contralateral hemispheres at Bregma −1.9 mm. One worksheet per mouse (n = 8). File: s7_7d_sham_sap102.xlsx

Data S8. SAP102 puncta measurements in LFPI mice at 28 days, related to Figures 2, S4, and S5 Per-region SAP102-mKO2 puncta count, mean punctum area, mean punctum intensity and proportion of puncta colocalised with PSD95-eGFP, in the ipsilateral and contralateral hemispheres at Bregma −1.9 mm. One worksheet per mouse (n = 10). File: s8_28d_injury_sap102.xlsx

Data S9. SAP102 puncta measurements in sham-LFPI mice at 28 days, related to Figures 2 and S5 Per-region SAP102-mKO2 puncta count, mean punctum area, mean punctum intensity and proportion of puncta colocalised with PSD95-eGFP, in the ipsilateral and contralateral hemispheres at Bregma −1.9 mm. One worksheet per mouse (n = 10). File: s9_28d_sham_sap102.xlsx

Data S10. SAP102 puncta measurements in surgery-naive mice, related to Figure S4 Per-region SAP102-mKO2 puncta count, mean punctum area, mean punctum intensity and proportion of puncta colocalised with PSD95-eGFP, in the ipsilateral and contralateral hemispheres at Bregma −1.9 mm. One worksheet per mouse (n = 5). File: s10_naive_sap102.xlsx

Data S11. Synapse subtype densities in LFPI mice at 7 days, related to Figures 1, 3, and S6 Per-region density of each of the 37 synapse subtypes (Subtype 01–37) and of puncta not assigned to a subtype (Subtype Others), in the ipsilateral and contralateral hemispheres at Bregma −1.9 mm. Subtypes 01–11 are Type 1 (PSD95-only), 12–19 are Type 2 (SAP102-only) and 20–37 are Type 3 (PSD95 and SAP102 colocalised). One worksheet per mouse (n = 11). File: s11_7d_injury_subtypes.xlsx

Data S12. Synapse subtype densities in sham-LFPI mice at 7 days, related to Figures 1, 3, and S6 Per-region density of each of the 37 synapse subtypes (Subtype 01–37) and of puncta not assigned to a subtype (Subtype Others), in the ipsilateral and contralateral hemispheres at Bregma −1.9 mm. Subtypes 01–11 are Type 1 (PSD95-only), 12–19 are Type 2 (SAP102-only) and 20–37 are Type 3 (PSD95 and SAP102 colocalised). One worksheet per mouse (n = 8). File: s12_7d_sham_subtypes.xlsx

Data S13. Synapse subtype densities in LFPI mice at 28 days, related to Figures 1, 3, and S6 Per-region density of each of the 37 synapse subtypes (Subtype 01–37) and of puncta not assigned to a subtype (Subtype Others), in the ipsilateral and contralateral hemispheres at Bregma −1.9 mm. Subtypes 01–11 are Type 1 (PSD95-only), 12–19 are Type 2 (SAP102-only) and 20–37 are Type 3 (PSD95 and SAP102 colocalised). One worksheet per mouse (n = 10). File: s13_28d_injury_subtypes.xlsx

Data S14. Synapse subtype densities in sham-LFPI mice at 28 days, related to Figures 1, 3, and S6 Per-region density of each of the 37 synapse subtypes (Subtype 01–37) and of puncta not assigned to a subtype (Subtype Others), in the ipsilateral and contralateral hemispheres at Bregma −1.9 mm. Subtypes 01–11 are Type 1 (PSD95-only), 12–19 are Type 2 (SAP102-only) and 20–37 are Type 3 (PSD95 and SAP102 colocalised). One worksheet per mouse (n = 10). File: s14_28d_sham_subtypes.xlsx

Data S15. Synapse subtype densities in surgery-naive mice, related to Figure 1 Per-region density of each of the 37 synapse subtypes (Subtype 01–37) and of puncta not assigned to a subtype (Subtype Others), in the ipsilateral and contralateral hemispheres at Bregma −1.9 mm. Subtypes 01–11 are Type 1 (PSD95-only), 12–19 are Type 2 (SAP102-only) and 20–37 are Type 3 (PSD95 and SAP102 colocalised). One worksheet per mouse (n = 5). File: s15_naive_subtypes.xlsx

Data S16. Microglial and integrated synapse metrics, related to Figure 4 IBA1 particle count, total labeled area, mean particle size and percentage area, together with PSD95 and SAP102 puncta density, colocalisation, synapse subtype densities and aggregate Type 1–3, SPL and LPL densities. Values are given for cortex, hippocampus, thalamus and hypothalamus in each hemisphere, with one row per mouse, region and hemisphere across all cohorts (surgery-naïve, n = 4; sham-LFPI 7 days, n = 8; sham-LFPI 28 days, n = 9; LFPI 7 days, n = 10; LFPI 28 days, n = 8). File: s16_Iba1_synapse_metrics.xlsx
