# Supplementary material for: Remodeling of synaptome architecture following traumatic brain injury
Source: iScience. 2026 Oct 29;29(11):118044. doi: 10.1016/j.isci.2026.118044 (PMC13742853; doi:10.1016/j.isci.2026.118044)
Supplement: Supplementary file 1 [file mmc1.pdf]

iScience, Volume *29*

Supplemental information

Remodeling of synaptome architecture following traumatic brain injury

Aimun A.B. Jamjoom, Ragini Gokhale, Jonathan Rhodes, Zhen Qiu, and Seth G.N. Grant

SUPPLEMENTARY MATERIAL

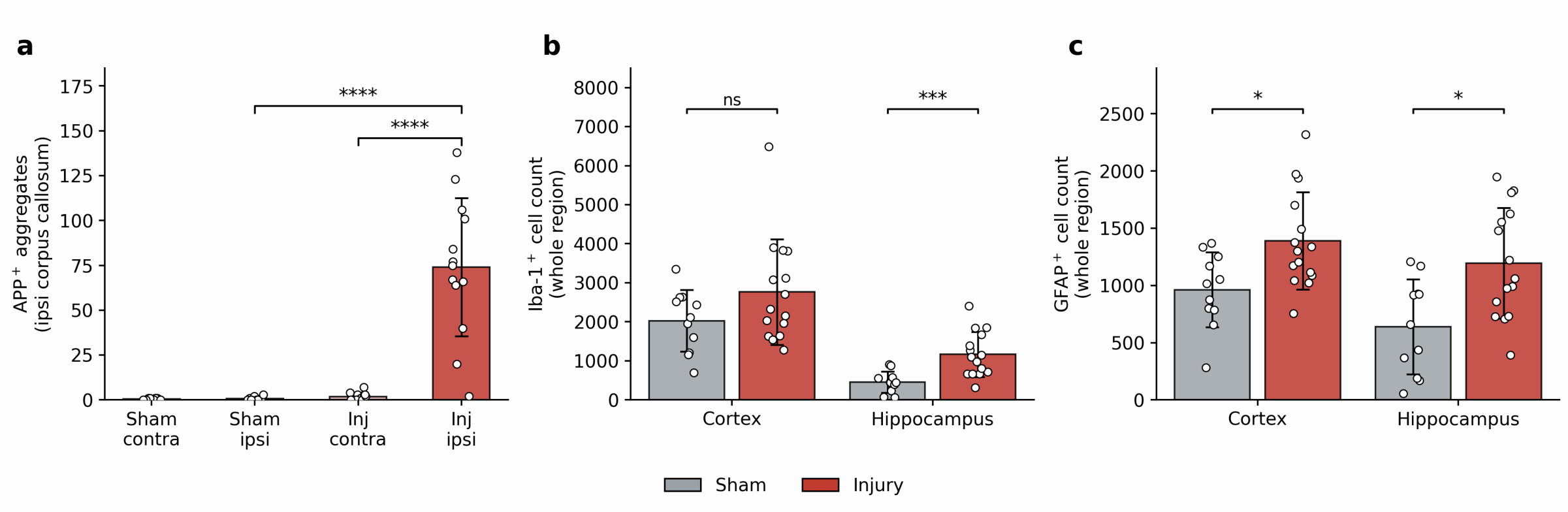

Figure S1: Characterization of Lateral Fluid Percussion Injury model

(a) Axonal injury indexed by amyloid precursor protein (APP) aggregates in ipsilateral corpus callosum.

(b) Microglia cell number in ipsilateral cortex and hippocampus.

(c) Astrocyte cell number in ipsilateral cortex and hippocampus.

Bars show mean ± SD; each point represents an individual mouse. *p<0.05; ***, p<0.001; ****, p<0.0001; ns, not significant. Sham, sham-LFPI; Inj, LFPI injury.

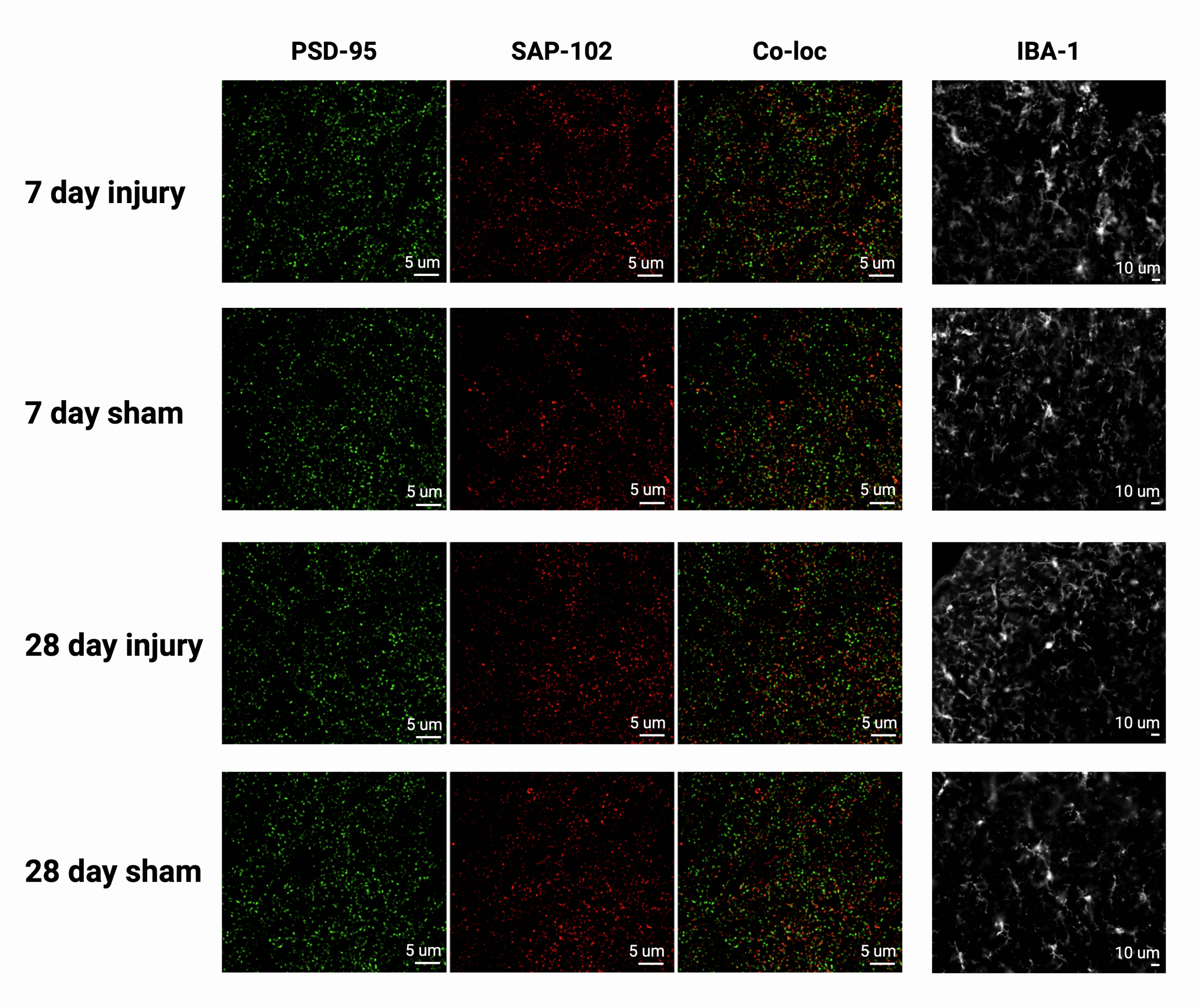

Figure S2. Representative images of synapses and microglia.

Synaptic puncta expressing PSD95-eGFP and/or SAP102-mKO2 in cortex at 7 and 28 days in injury and sham mice. Microglial cells labelled with IBA-1. Scale bars are present on all representative micrographs (5 μm for the PSD95, SAP102 and co-localisation panels; 10 μm for the IBA1 panels).

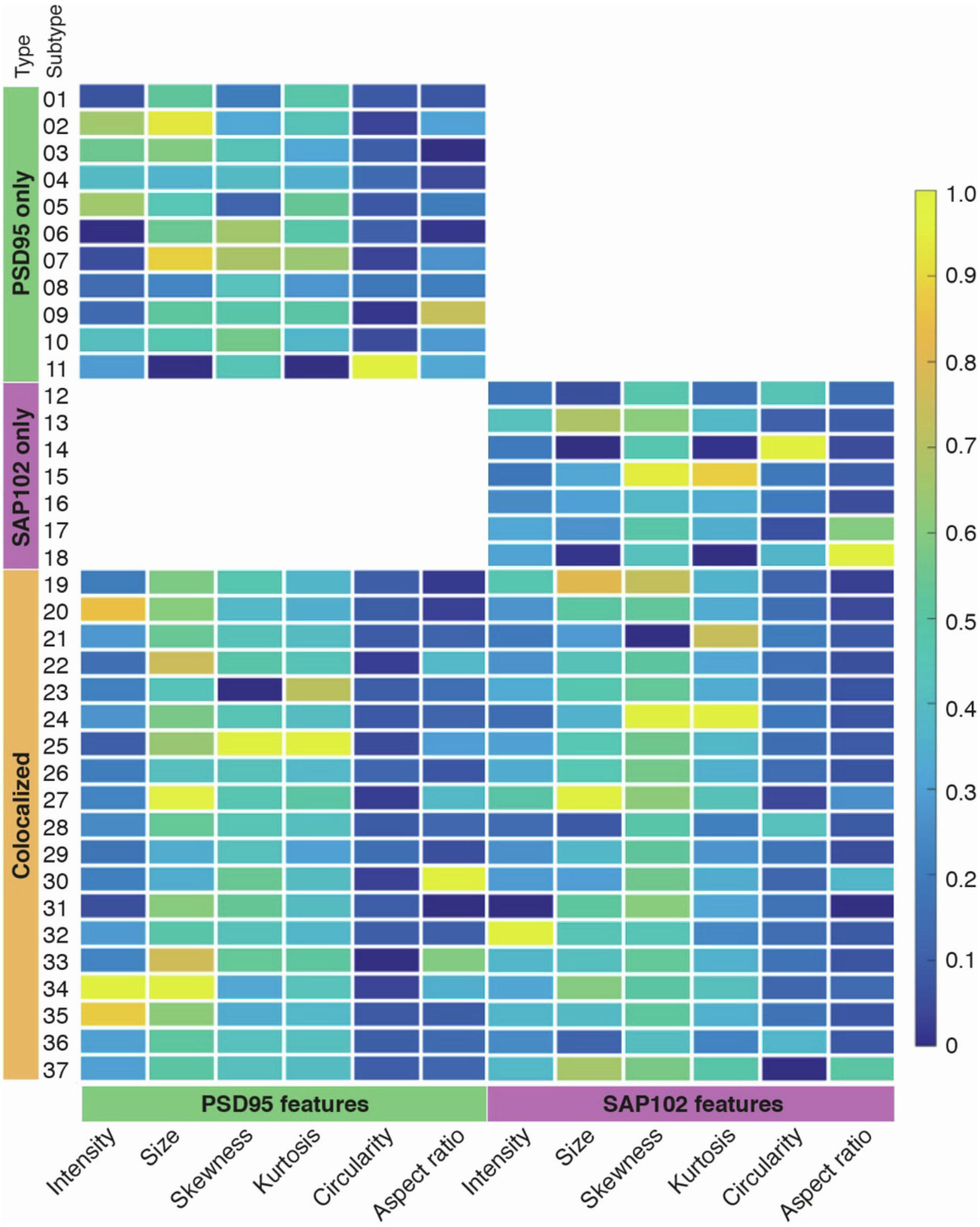

Figure S3. Catalog of the 37 synapse subtypes and their parameters.

Median value of the synaptome parameters (intensity, size, skewness, kurtosis, circularity, aspect ratio) for PSD95 (left side) and SAP102 (right side), for each of three types of synapses (PSD95-only; SAP102-only; Colocalized) that are further divided into 37 subtypes. Values are normalized for each parameter. Reproduced from Figure S9 of Zhu et al, 2018 [S1].

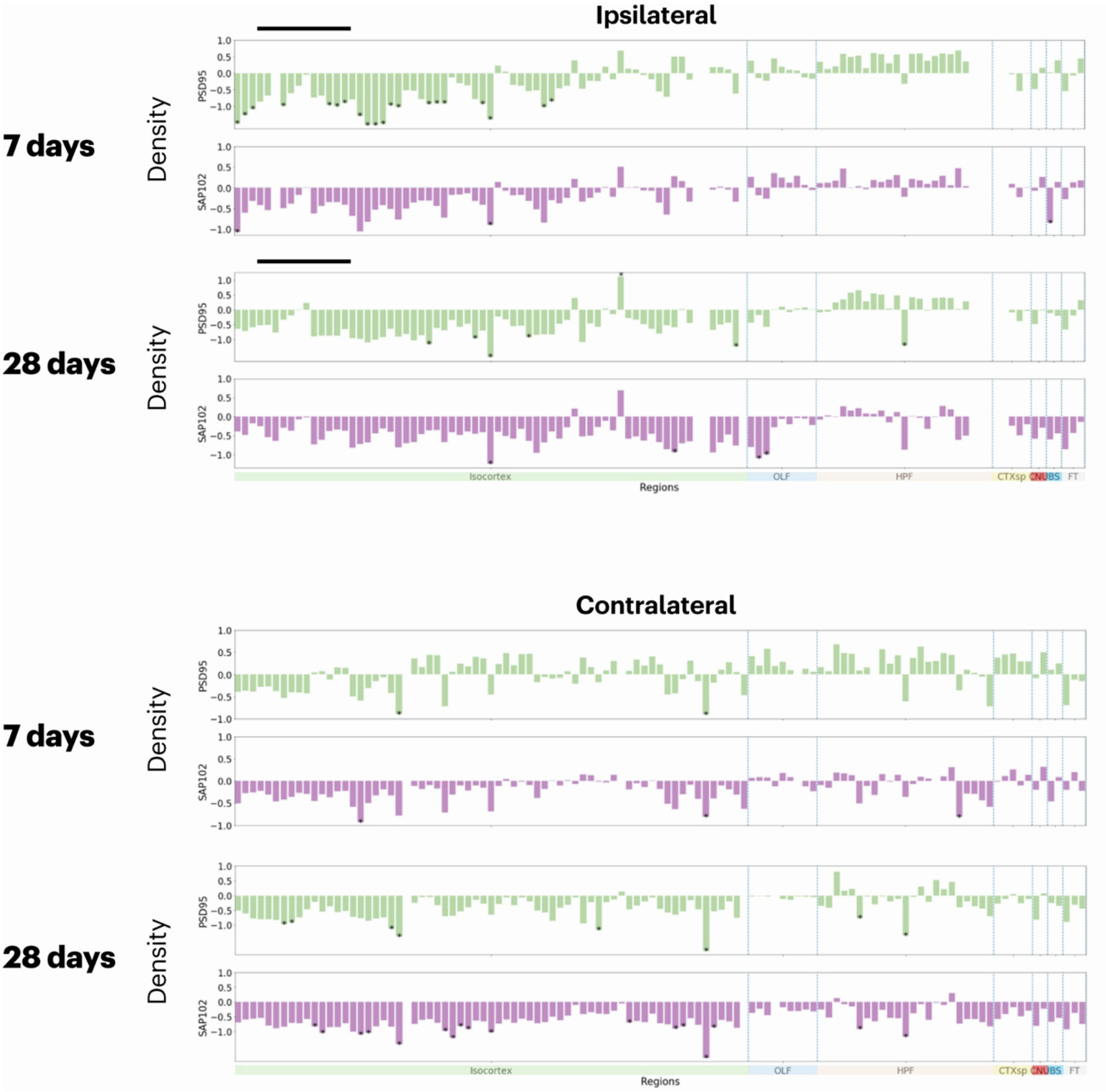

Figure S4: Effect size of PSD95 and SAP102 density between injury and surgery-naïve mice

Cohen's *d* effect size of puncta density between injury (LFPI) and surgery-naïve groups at 7D and 28D (*$p<0.05$, Bayesian test with Benjamini–Hochberg correction). Black line, location of injury site.

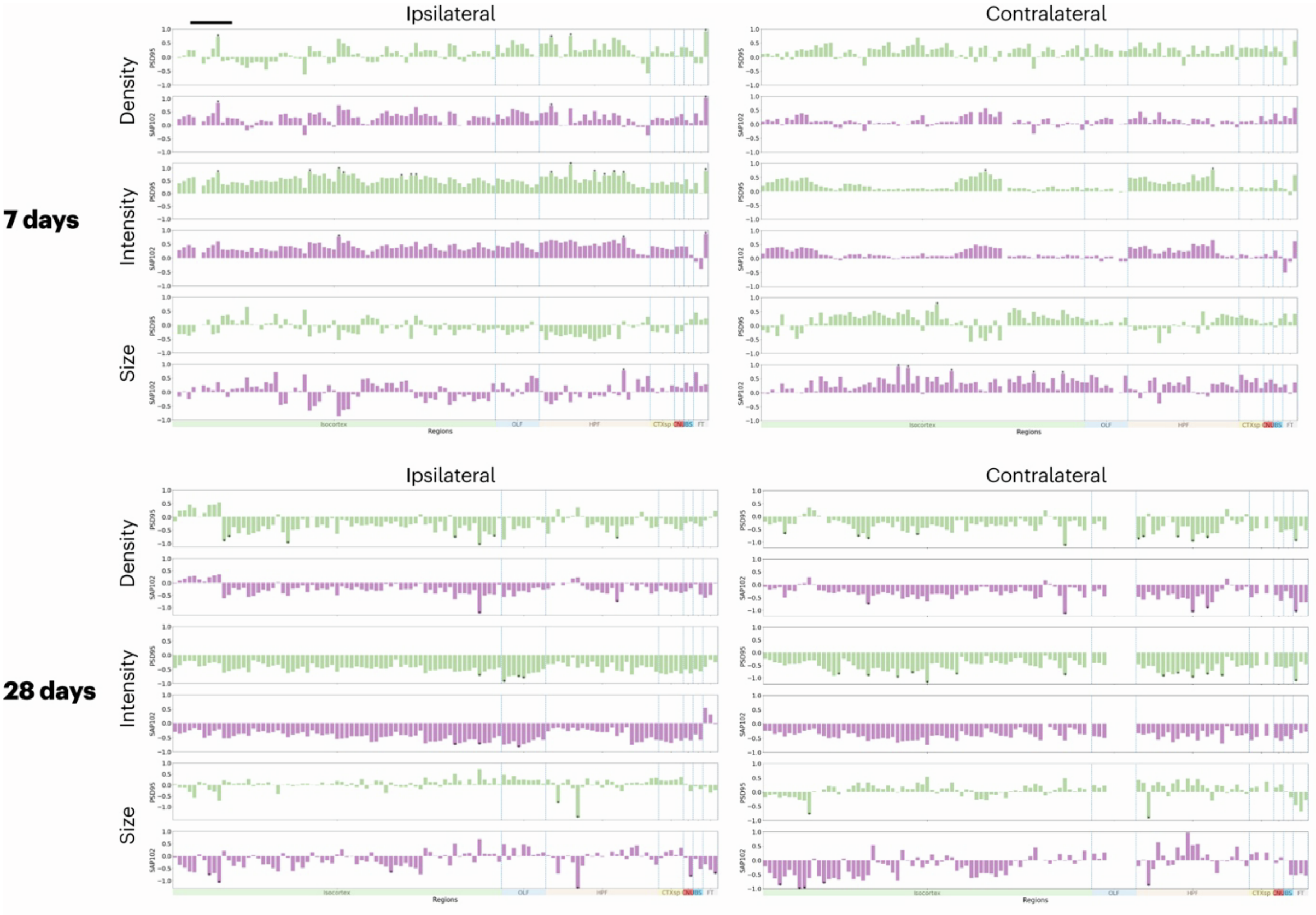

Figure S5: Effect size of PSD95 and SAP102 density, intensity and size between injury and sham mice

Cohen's *d* effect size of puncta density, intensity and size between injury (LFPI) and sham-LFPI groups at 7D and 28D (*$p<0.05$, Bayesian test with Benjamini–Hochberg correction). Black line above 7D ipsilateral data shows location of injury site.

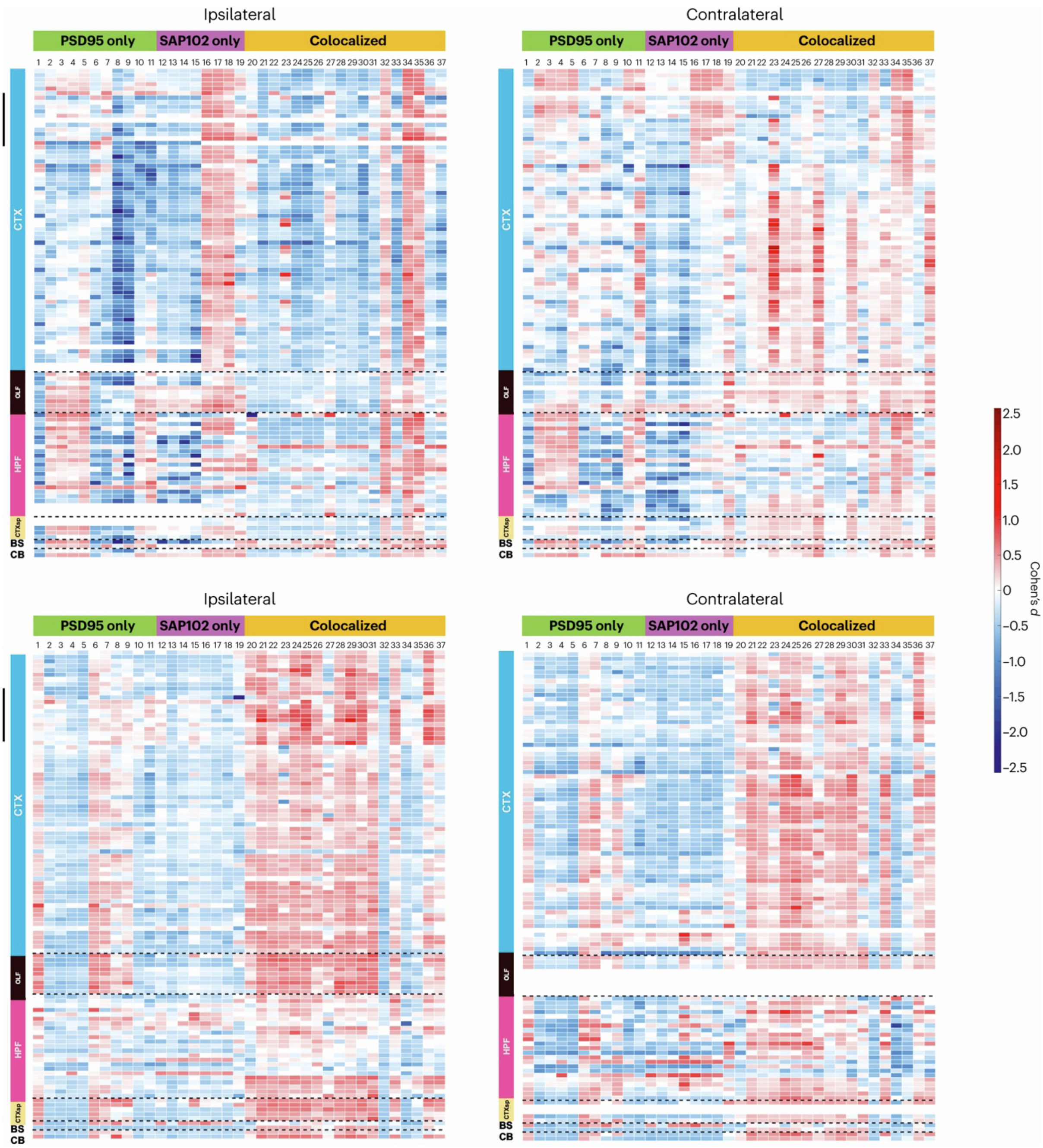

Figure S6: Heatmaps of effect sizes comparing injury and sham mice categorised by synapse subtypes

Heatmap of Cohen's *d* effect size (key on right of each plot) of puncta density between injury (LFPI) and sham-LFPI groups at 7D (top panels) and 28D (bottom panels) categorised by synapse type and subtype. Black line on left of heat maps shows location of injury site.

Supplemental references

1) Zhu, F. *et al*. Architecture of the Mouse Brain Synaptome. *Neuron* **99**, 781-799 e710 (2018). https://doi.org/10.1016/j.neuron.2018.07.007
